# Hybrid Open Anterior and Laparoscopic Repair Using Self-Gripping Mesh for Parastomal Hernia Following Ileal Conduit With Extensive Intra-abdominal Adhesions: A Case Report

**DOI:** 10.7759/cureus.82500

**Published:** 2025-04-18

**Authors:** Yoh Kitamura, Shingo Tsujinaka, Yoshihiro Sato, Tomoya Miura, Chikashi Shibata

**Affiliations:** 1 Gastroenterologic Surgery, Tohoku Medical and Pharmaceutical University, Sendai, JPN

**Keywords:** ileal conduit, onlay mesh placement, parastomal hernia, self-gripping mesh, small bowel obstruction

## Abstract

Parastomal hernia (PSH) is the protrusion of visceral organs through an abdominal wall defect adjacent to a stoma and is one of the major complications following cystectomy and ileal conduit (IC) formation. We report a case of hybrid open anterior and laparoscopic repair using self-gripping mesh for a PSH following IC, complicated by extensive intra-abdominal adhesions.

An 89-year-old man presented with recurrent episodes of small bowel obstruction (SBO) caused by PSH following IC. The patient had undergone total cystectomy with IC for urinary bladder cancer 30 years prior and had been hospitalized nine times for SBO due to PSH. The patient was referred for surgical treatment. Computed tomography revealed protrusion of the small bowel through a 10 × 7 cm hernia orifice around the IC. Considering the symptomatic PSH with a persistent risk of SBO, laparoscopic repair was planned. Laparoscopic exploration revealed extensive adhesions of the small bowel to the hernia orifice and IC, extending to the pelvis. The IC was also widely attached to the anterior abdominal wall, preventing visual assessment of the contralateral side of the conduit. Therefore, an additional transverse skin incision was made laterally and caudally to the stoma. The defect was closed anteriorly under direct vision with interrupted transfascial sutures and reinforced by onlay mesh placement using a trimmed (15 × 12 cm) self-gripping mesh (ProgripTM, Medtronic). The postoperative course was uneventful. At the 15-month follow-up, the patient was in good physical condition without hernia recurrence or SBO, except for intermittent episodes of urinary obstruction requiring drainage.

Hybrid open anterior and laparoscopic repair using self-gripping mesh may be considered a surgical option for PSH following IC with extensive intra-abdominal adhesions around the stoma.

## Introduction

Parastomal hernia (PSH) is the protrusion of visceral organs through an abdominal wall defect adjacent to a stoma and is one of the major complications following cystectomy and ileal conduit (IC) formation, with incidence rates reported in the literature ranging from 17% to 30% [1-3]. Symptoms of PSH can vary from mild discomfort to severe, potentially life-threatening conditions, including intense abdominal pain, bowel obstruction, and perforation [3-5]. Although most patients with PSH can be managed nonoperatively, surgical intervention may be required for those experiencing intractable skin irritation, difficulty in sealing the pouch, or previously mentioned life-threatening conditions [3,4]. Surgical treatments for PSH include stoma relocation, direct repair, onlay mesh repair, retromuscular repair, 3D funnel-shaped mesh repair, and intraperitoneal onlay mesh (IPOM) repair [1-3,5-9]. The evolution of surgical approaches has progressed from open laparotomy to laparoscopic and robotic-assisted surgery [3,5-9]. IPOM repair is represented by the Sugarbaker (SB) and the keyhole (KH) techniques, with recent evidence favoring the SB due to its lower recurrence rate compared to other methods [6-9].

Despite advancements, high recurrence rates - ranging from 0% to 80%, and 23% to 47% in larger case series - remain a significant surgical challenge [3,5-10]. A recent comparative study showed that PSH following IC is associated with more severe intra-abdominal adhesions compared to those following colostomy, resulting in greater surgical difficulty [10]. Moreover, a high prevalence of severe adhesions has been observed in patients with recurrent PSH, with approximately 40% of these individuals undergoing another surgery due to complications after recurrent PSH repair [7]. To avoid these devastating consequences, careful surgical planning, along with appropriate intraoperative assessment and decision-making, is crucial.

To address intra-abdominal adhesions, a hybrid approach combining laparoscopic adhesiolysis and open IPOM repair has recently been proposed, demonstrating a low recurrence rate of 4% [11]. Moreover, onlay mesh repair for PSH using a lateral peristomal incision, rather than a midline incision, remains an alternative option for high-risk patients with a low morbidity rate (11%) and an acceptable recurrence rate (21%) [12].

Herein, we report a case of hybrid open anterior and laparoscopic repair using self-gripping mesh for a PSH following IC, complicated by extensive intra-abdominal adhesions.

## Case presentation

An 89-year-old man presented with recurrent episodes of small bowel obstruction (SBO) caused by a PSH following an IC. The patient had undergone total cystectomy with IC for urinary bladder cancer 30 years prior. Within a year of the surgery, he developed a bulge and pain around the stoma and was diagnosed with PSH. Since then, he had been hospitalized nine times for SBO due to PSH, with the most recent three hospitalizations occurring within the same year. The most relevant symptoms included a bulge, abdominal pain, and urinary obstruction requiring catheterization into the IC. As the intervals between hospitalizations gradually shortened, the patient was referred for surgical treatment.

**Figure 1:**
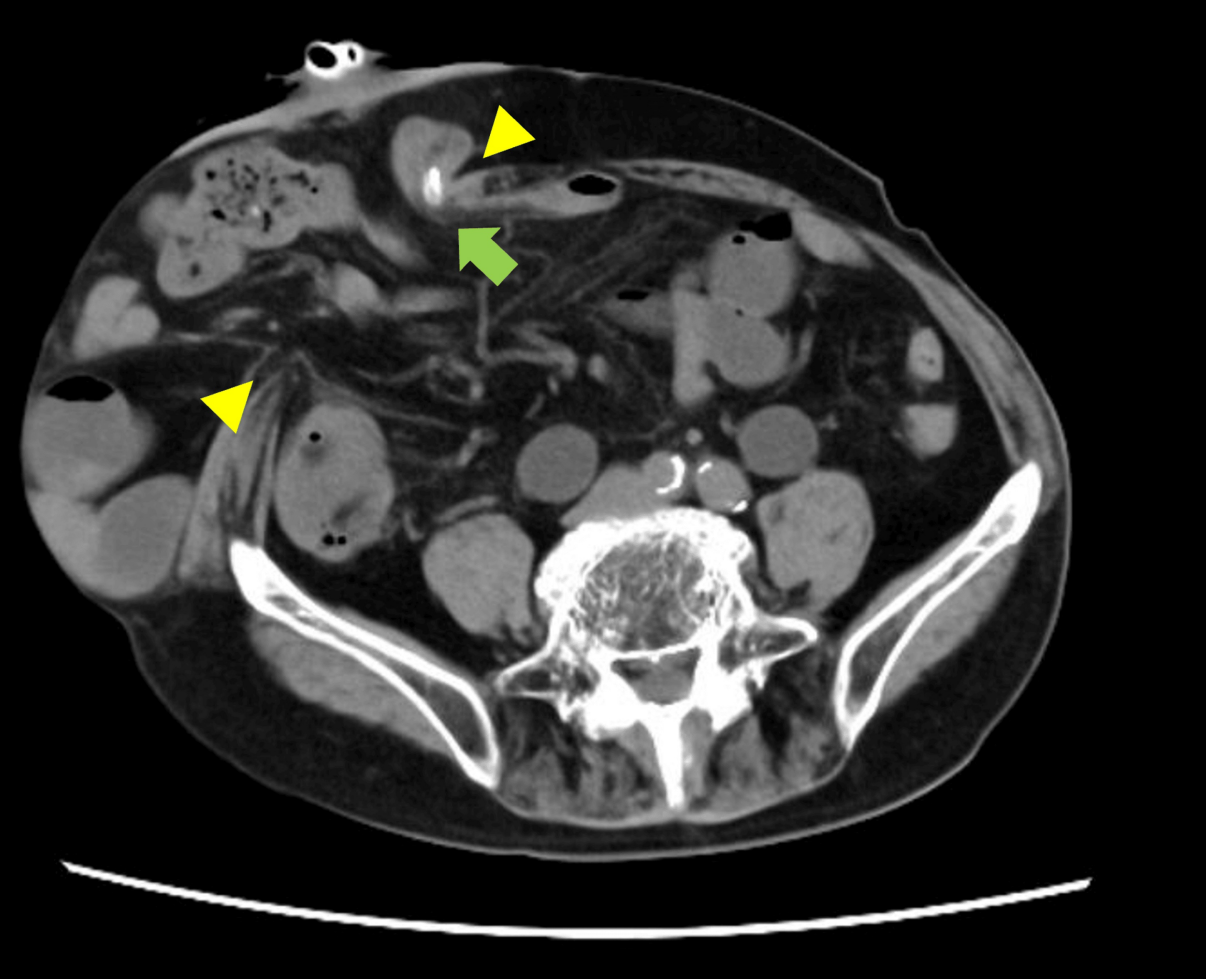
Preoperative computed tomography (CT): Axial view CT shows protrusion of the small bowel through the hernia orifice. The width of the orifice is 10 cm (yellow arrowheads). The ileal conduit (IC) is located at the internal edge of the hernia orifice, and a urinary catheter is placed for concomitant urinary obstruction (green arrow).

**Figure 2:**
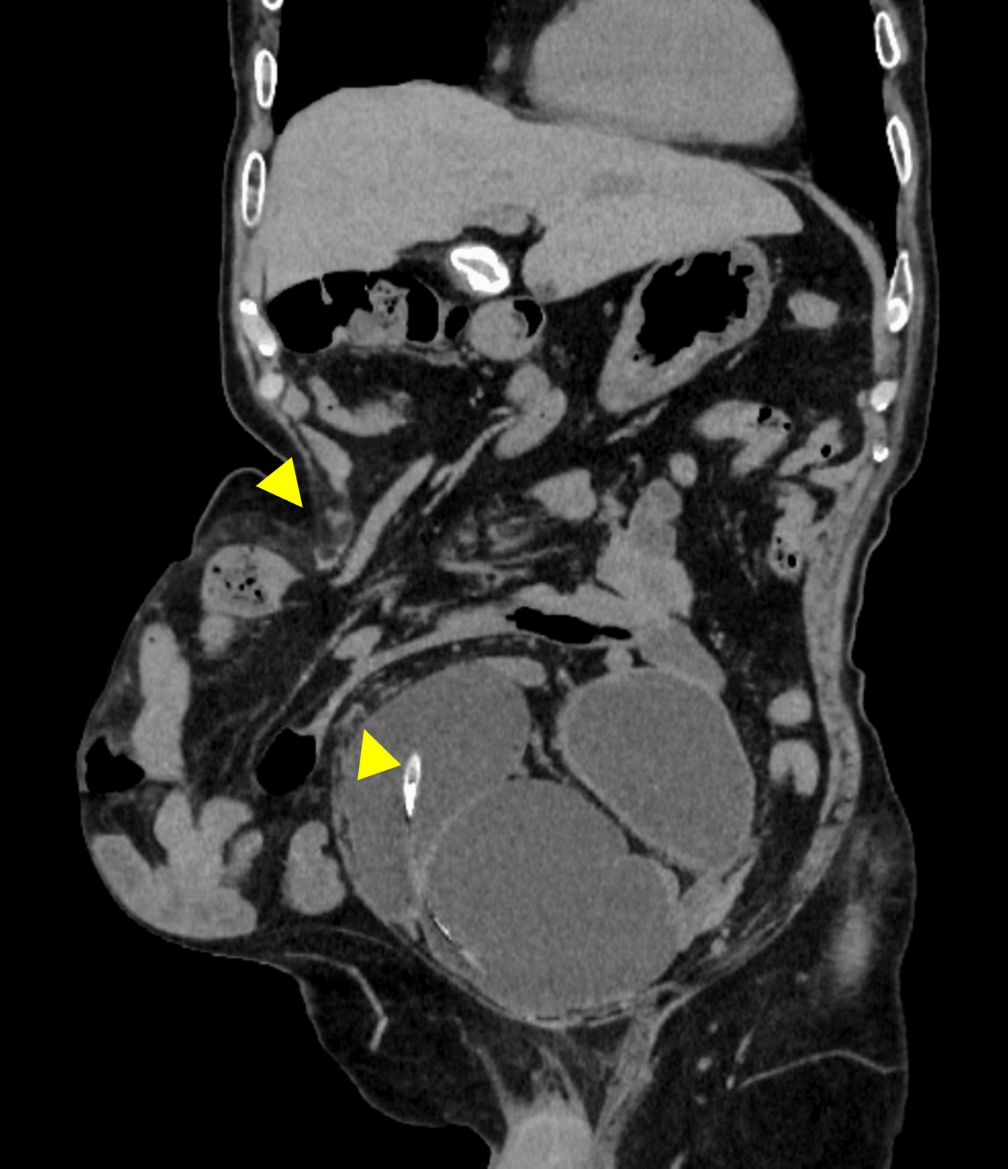
Preoperative computed tomography (CT): Semi-coronal view CT shows protrusion of the small bowel through the hernia orifice. The width of the orifice is 10 cm (yellow arrowheads).

Computed tomography (CT) revealed a protrusion of the small bowel through a 10 × 7 cm hernia orifice around the IC (Figures 1-2), which was categorized as type III PSH (defect size: larger than 5 cm, without concomitant incisional hernia) according the definition of the European Hernia Society [13]. 

Considering the symptomatic PSH with a persistent risk of SBO, laparoscopic repair was planned.

**Figure 3:**
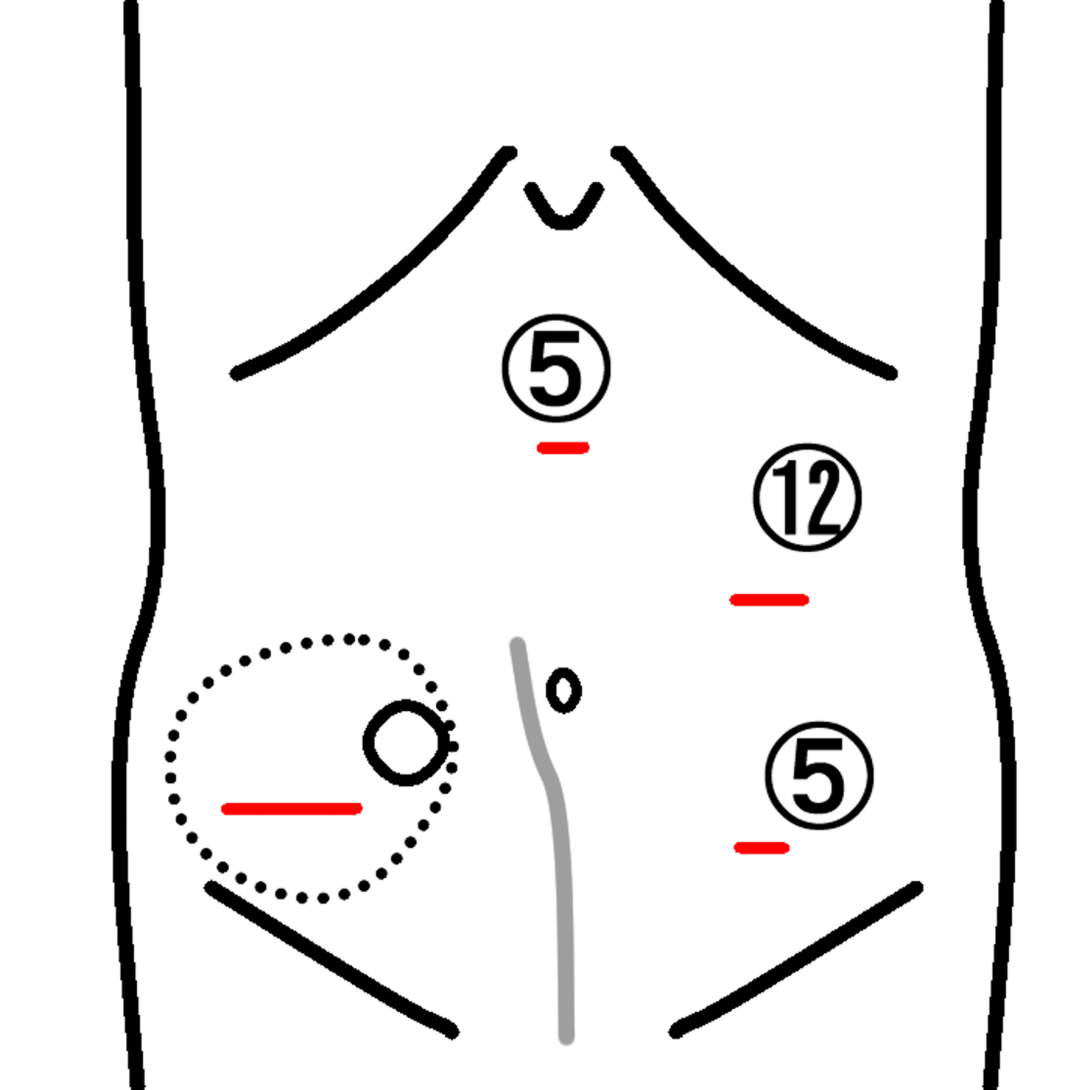
Position of working ports and skin incision Schematic presentation of the hernia location (dotted line), opening of the ileal conduit (circle), previous surgical scar (gray line), the wounds with the size of working ports (mm), and the additional skin incision made caudally and laterally to the stoma (transverse red lines). Source: This is our artwork, created using Microsoft PowerPoint software (Microsoft Corp., Redmond, WA, USA).

Under general anesthesia, the patient was placed in the supine position. Port placement was performed as shown in Figure 3.

**Figure 4:**
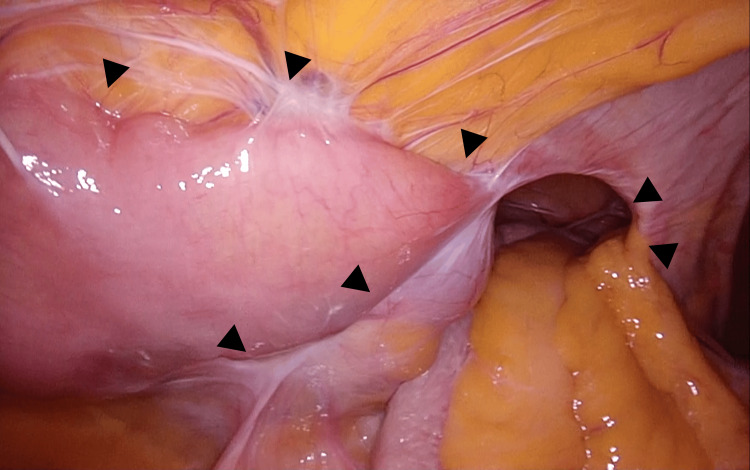
Intra-abdominal adhesions Laparoscopic exploration revealed extensive adhesions of the small bowel to the hernia orifice and ileal conduit, extending to the pelvis along the previous midline incision (black arrowheads).

The procedure commenced with the insertion of a 12 mm port using the open Hasson technique in the left abdomen. Next, 5 mm ports were placed in the upper and lower abdomen. Laparoscopic exploration revealed extensive adhesions of the small bowel to the hernia orifice and IC, extending to the pelvis along the previous midline incision (Figure 4).

**Figure 5:**
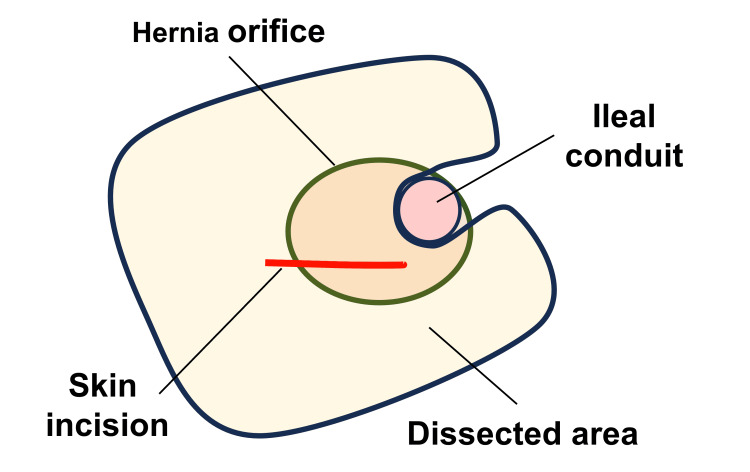
Exposure of the hernia orifice Schematic presentation shows the location of the ileal conduit, hernia orifice, transverse skin incision, and the dissected area for mesh placement. Source: This is our artwork, created using Microsoft PowerPoint software (Microsoft Corp., Redmond, WA, USA).

**Figure 6:**
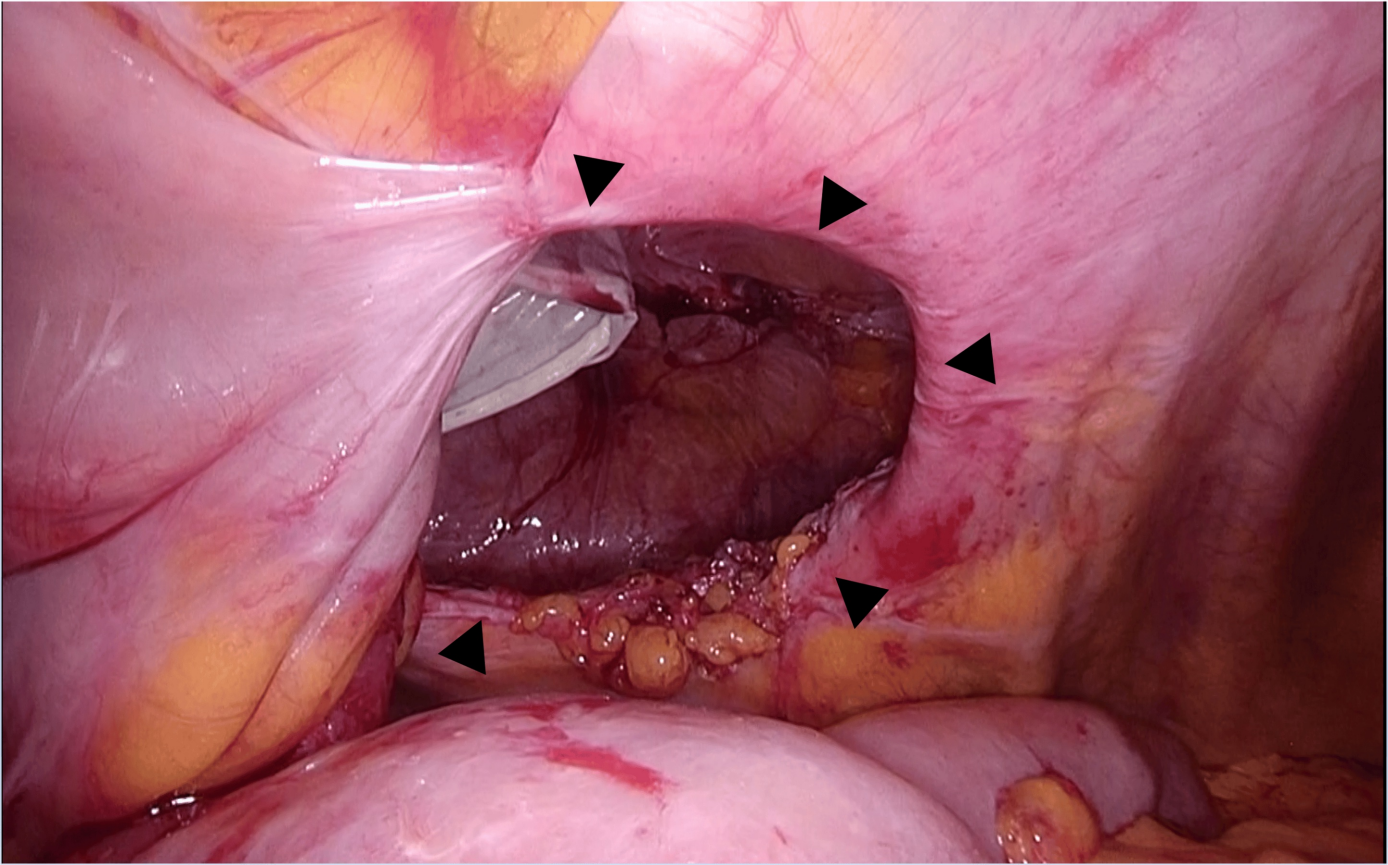
Exposure of the hernia orifice Laparoscopic view of the hernia orifice, measuring 8 × 6 cm in diameter, exposed after adhesiolysis (black arrowheads).

The IC was also widely attached to the anterior abdominal wall, preventing visual assessment of the contralateral side of the conduit. Therefore, an additional transverse skin incision was made laterally and caudally to the stoma (Figure 2). Subsequently, the hernia sac was opened and excised, and the adhesions around the IC were detached under direct vision. Finally, the hernia orifice, located laterally to the IC and measuring 8 × 6 cm in diameter, was exposed (Figures 5-6).

**Figure 7:**
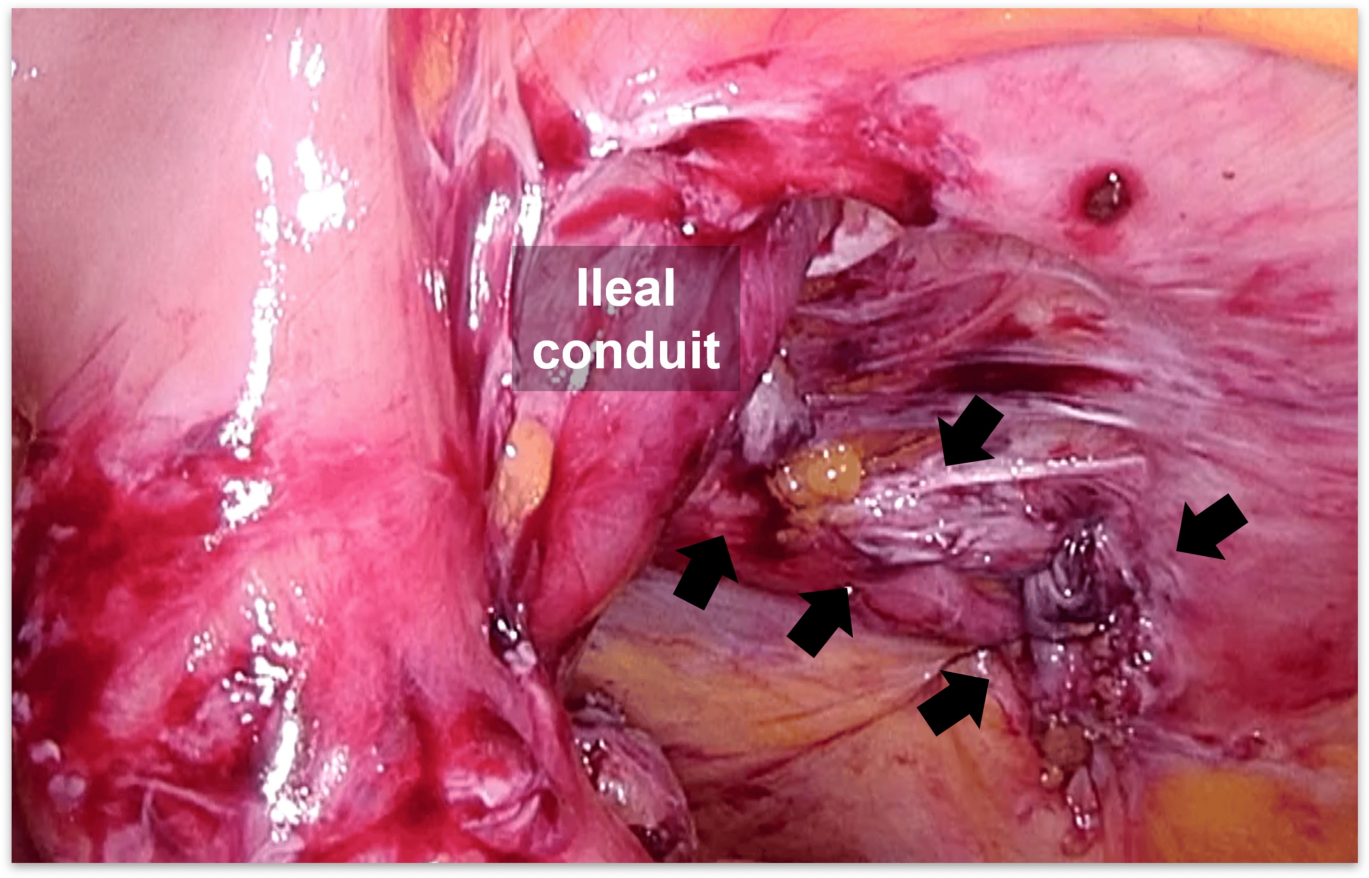
Laparoscopic view of the closed hernia orifice The defect was closed anteriorly under direct vision (black arrows) to avoid the risk of unintended bowel injury due to intra-abdominal adhesions.

**Figure 8:**
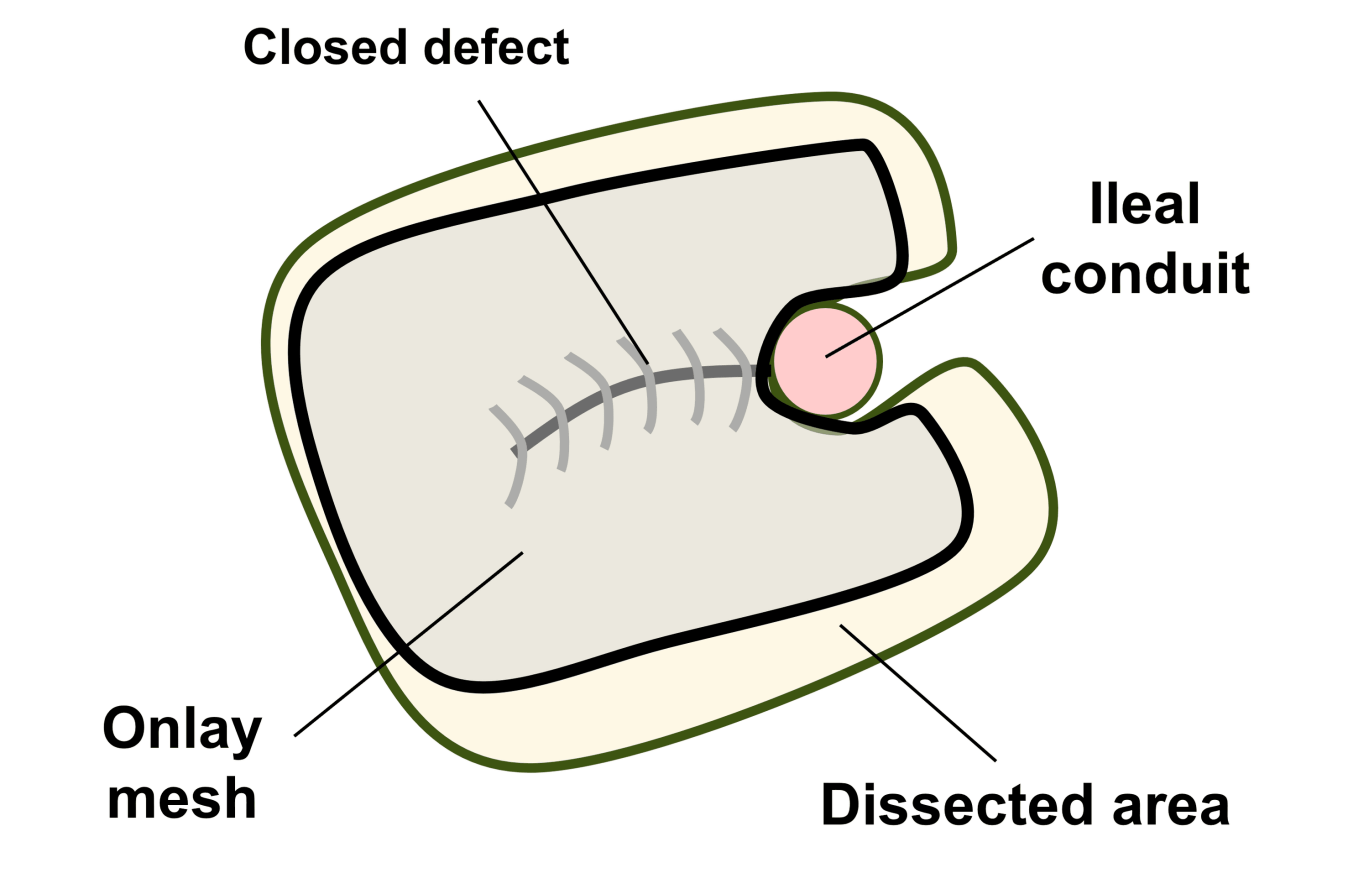
Stoma location and the onlay mesh Schematic presentation shows the location of the ileal conduit, closed defect, onlay mesh, and dissected area for mesh placement. Source: This is our artwork, created using Microsoft PowerPoint software (Microsoft Corp., Redmond, WA, USA).

**Figure 9:**
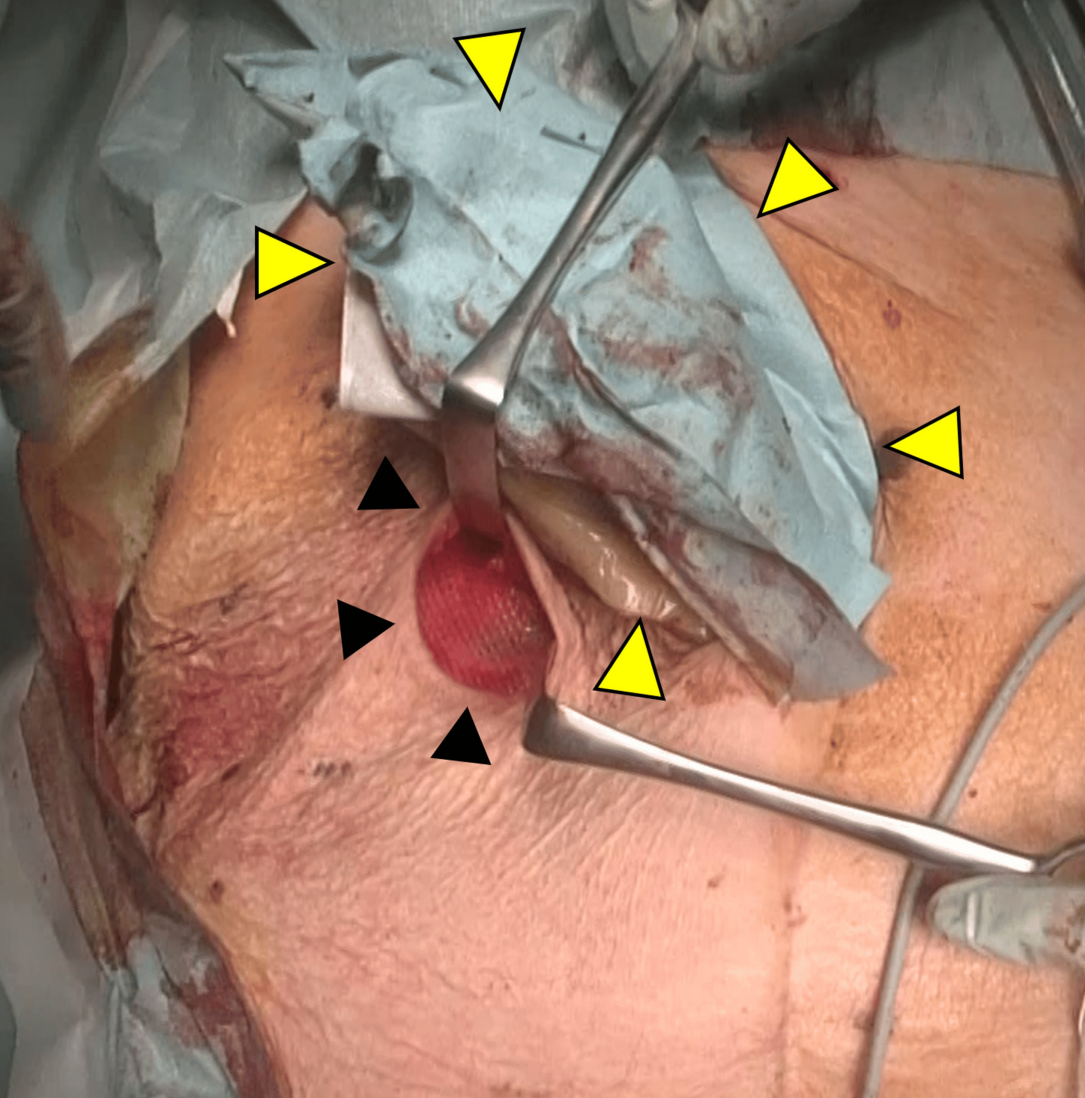
Stoma location and the onlay mesh The onlay mesh was placed beneath the skin incision (black arrowheads). The stoma was protected using surgical drapes during the procedure (yellow arrowheads).

Considering the remaining extensive intra-abdominal adhesions, the defect was closed anteriorly under direct vision with interrupted transfascial sutures using 0-PolysorbTM (Medtronic, Fridley, MN, USA). Laparoscopic inspection confirmed complete closure of the hernia orifice (Figure 7), and an anti-adhesive agent (AdsprayTM, Terumo Inc., Tokyo, Japan) was disseminated intra-abdominally around the IC. The subcutaneous area was further dissected to expose the anterior rectus sheath, and the closed defect was then reinforced by onlay mesh placement using a trimmed (15 × 12 cm) self-gripping mesh (ProgripTM, Medtronic) (Figures 8-9).

All skin incisions were closed with subcuticular sutures. The operative time was 223 minutes, and the estimated blood loss was 30 mL.

The postoperative course was uneventful, except for the development of a subcutaneous seroma, which spontaneously resolved four months postoperatively. At 15-month follow-up, the patient was in good physical condition without hernia recurrence or SBO, except for intermittent episodes of urinary obstruction requiring drainage.

## Discussion

In our case, extensive intra-abdominal adhesions necessitated conversion from purely laparoscopic repair to a hybrid open anterior and laparoscopic approach. Previous literature has also identified severe adhesions as a major factor contributing to conversion to laparotomy. Suwa et al. reported that two out of ten cases of PSH following IC required conversion to laparotomy [10]. The reasons for conversion included ureteral injury during adhesiolysis from the lateral abdominal wall and strong adhesion of the cecum to the hernia sac. A more recent study revealed that 63% of patients with recurrent PSH had perioperative severe adhesions [7]. Traumatic adhesiolysis can result in intraoperative contamination from inadvertent bowel injury, potentially necessitating subsequent mesh removal [7,14]. Additionally, difficult adhesiolysis may restrict the use of synthetic mesh for recurrent PSH repair and become a primary cause of further interventions [7]. Therefore, in cases of severe or extensive adhesions around the IC, the IPOM technique, including the SB or KH, may not be feasibly indicated. Instead, direct or onlay repair may be preferred; however, the higher recurrence rates reported for these methods compared to sublay or IPOM repair may limit their viability as alternative treatment options [3,5,6].

A self-gripping mesh is a synthetic prosthetic material designed to provide secure reinforcement without the need for additional fixation sutures or devices such as tackers [15]. It has been shown to facilitate a safe and durable tension-free repair in both the open onlay technique [16] and the sublay technique [17] for abdominal incisional hernia. Various mesh-related complications, including seroma and/or hematoma, have been reported in up to 28.1% of cases; however, hernia recurrence rates in these reports were relatively low, ranging from 0% to 8.1% [16,17]. To the best of our knowledge, this is the first report to utilize a self-gripping mesh for the treatment of PSH using any repair technique. Meticulous care should be taken to avoid injury to adjacent visceral organs during the procedure in order to prevent mesh contamination. In our case, a drain was not placed to avoid interfering with stoma care, which resulted in the development of a subcutaneous seroma. The use of suction drains can help prevent fluid collection and subsequent infection [17]; therefore, a drain could potentially have been placed at a distant site from the wound for a short period.

Szczepkowski et al. recently proposed a hybrid PSH endoscopic repair (HyPER) technique for PSH [11]. The procedure comprises four key stages: first, laparoscopic complete adhesiolysis; second, dissection of the hernia sac, intraperitoneal placement of mesh, and narrowing of the trephine using non-absorbable sutures via an open anterior approach; third, laparoscopic fixation of the intraperitoneal mesh with tackers; and fourth, open stoma formation and drain placement. Among 95 patients, the overall complication rate was 19%, including surgical site infection, hematoma, partial ostomy necrosis, and postoperative ileus. Most of these complications were managed conservatively without the need for surgical intervention. The mesh used was either synthetic or biological, and hernia recurrence occurred in four patients [11]. The authors suggested that the advantages of the HyPER technique include reliable excision of the hernia sac, narrowing of the hernia orifice, and fixation of a flat mesh in place [11]. In our case, the surgical concept and procedure were similar, but we abandoned complete adhesiolysis due to the risk of multiple bowel injuries, which could have limited the use of mesh, and the concern over prolonged operative time in an elderly patient. For these reasons, we selected an onlay repair using a self-gripping mesh in a hybrid open anterior and laparoscopic approach.

## Conclusions

Hybrid open anterior and laparoscopic repair may be considered one of the available surgical options for PSH following IC with extensive intra-abdominal adhesions around the stoma. A self-gripping mesh can be used to reinforce the defect closure. Further evaluation with a larger patient cohort and longer follow-up is necessary to assess the efficacy of self-gripping mesh in PSH repair.
